# Multimodal perioperative care plus immunonutrition versus traditional care in total hip arthroplasty: a randomized pilot study

**DOI:** 10.1186/s12937-016-0153-1

**Published:** 2016-04-02

**Authors:** Miguel Aprelino Alito, José Eduardo de Aguilar-Nascimento

**Affiliations:** 1 Brazilian Society of Orthopedics and Traumatology, Cuiabá, MT Brazil; 2 Federal University of Mato Grosso, Cuiabá, Brazil, Cuiabá, Brazil; 3 UNIVAG, Varzea Geande, Brazil; 4 Rodovia Helder Candia, Cond. Country casa 15, 78048-150 Cuiabá, Brazil

**Keywords:** Total hip arthroplasty, Perioperative care, C-reactive protein, Preoperative fasting, Immune nutrition, Length of hospital stay

## Abstract

**Background:**

Multimodal protocols of perioperative care may enhance postoperative recovery. However, limited information is available on preoperative immune and carbohydrate (CHO)-enriched drinks in patients undergoing hip arthroplasty. We aimed to investigate the effect of a multimodal protocol (ACERTO protocol) plus preoperative immune nutrition on the length of stay (LOS) and the postoperative acute phase response of patients undergoing total hip arthroplasty.

**Methods:**

Thirty-two patients (mean age, 58 years; range, 26–85 years; 16 males) were randomized to receive either the ACERTO protocol (*n* = 15, ACERTO Group), which consisted of 6 h preoperative fasting for solids, an oral drink (200 mL of 12.5 % maltodextrin) up to 2 h before induction of anesthesia, restricted intravenous fluids (only 1000 mL of crystalloid fluid after surgery) and preoperative immune nutrition (600 mL/day of Impact - Nestlé, Brazil) for five days prior to surgery, or traditional care (*n* = 17; control group), which consisted of 6–8 h preoperative fasting, intravenous hydration until the 1^st^ postoperative day and no preoperative immune supplementation. The main endpoint was LOS. C-reactive protein (CRP) was the secondary endpoint and was assessed during induction of anesthesia and on postoperative day 2.

**Results:**

Neither deaths nor postoperative complications occurred. The median LOS was 3 (2–5) days in the ACERTO group and 6 (3–8) days in controls (*P* <0.01). Postoperative CRP was higher in the control group (*P* <0.01).

**Conclusion:**

The ACERTO multimodal protocol of perioperative care plus preoperative immune nutrition may decrease LOS and postoperative CRP levels in total hip arthroplasty.

**Trial registration:**

Clinical Trials: NCT02580214

## Background

Total hip arthroplasty (THA) is a common orthopedic procedure and may improve long-term quality of life for many patients. More than 70,000 THA are performed each year in UK [1]. In the USA it is estimated that nearly 500,000 THA are performed each year [2] and that the demand for primary THA will increase by 174 % by the year 2030 [3]. As the hospital costs are high, solutions for decreasing complications and length of stay are demanded.

A review of the evidence has shown that traditional perioperative care has a weak scientific basis and needs to be changed [4]. Multimodal or fast-track protocols of perioperative care for abdominal surgery have been associated with lower morbidity, lower costs and faster postoperative recovery than traditional care [5]. Similarly, the use of oral supplements containing immune nutrients such as arginine and omega-3 fatty acids for 5–7 days before major abdominal surgery may decrease the risk of infection and reduce postoperative length of stay (LOS) [6]. Of note, studies of the effect of multimodal protocols, including immune nutrition, on postoperative recovery from elective THA are lacking [7]. Probable barriers to implementation involve multiple care disciplines [8]. Although the outcome after THA has improved over time, in parallel with the development of improved surgical techniques and pain control, we assume that the use of a multimodal protocol that includes preoperative immune nutrition may enhance postoperative recovery [9]. In Brazil, the ACERTO (an acronym in Portuguese for acceleration of postoperative recovery) protocol is a multimodal protocol of perioperative care that includes shorter preoperative fasting with carbohydrates (CHO)-rich drinks up to 2 h before surgery, restriction of intravenous fluids, early postoperative feeding and early mobilization [10, 11]. Recently, the multimodal ACERTO protocol added a recommendation of perioperative immune nutrition. All the elements of the protocol are evidence-based and are recommended by several international guidelines and consensus panels. Thus, the aim of this pilot study was to investigate whether the use of the ACERTO multimodal protocol plus preoperative immune nutrition would decrease the LOS and acute phase inflammation in patients who undergo THA.

## Methods

This was a prospective, randomized pilot study. All procedures were performed at the São Mateus Hospital (Cuiabá, Brazil) from May 2012 to February 2013. The study was approved by the Research Ethics Committee of the Júlio Müller University Hospital (Federal University of Mato Grosso) and all patients signed an informed consent form. The trial was registered at Clinicaltrials.org with the number NCT02580214.

### Inclusion and exclusion criteria

We included adult patients (18–80 y/o) of both sexes who had hip osteoarthrosis and were candidates for elective THA. Patients were excluded if they had fasting glycemia measurements >200 mg/dL; acquired immunodeficiency; renal failure (creatinine >2 mg/dL); cirrhosis; moderate or severe Alzheimer's disease (clinical dementia rating score between 2 and 3); an American Society of Anesthesiologists (ASA) score >2; previous spinal surgery (arthrodesis) or previous THA (reviewing or changing the prostheses); or severe malnutrition (loss of 10 % of body weight over the last 6 months). We also excluded patients whose blood samples were not obtained at the scheduled time or who did not complete the perioperative protocol, e.g., did not consume the immune supplement if assigned to the ACERTO group.

### Study design

Patients were enrolled consecutively and randomized into two groups, control group and ACERTO group, using electronically generated random numbers available at www.graphpad.com. The main differences between the two groups in perioperative care are displayed in Table 1. Not only the surgeon but a multidisciplinary team saw all patients before the THA: a dietitian assessed anthropometric data and confirm the nutrition status, a nurse gave preoperative counseling and the anesthetist performed preoperative visit.

**Table 1:** Comparison of the perioperative care of the two study groups

	Control group	ACERTO group
Preoperative information	Yes	Yes
Preoperative fasting:	6–8 h fast prior to surgery.	6–8 h fast for solids; carbohydrate drink (12 % maltodextrin), 200 mL up to 2 h before surgery.
Preoperative nutrition	None	Immune supplement 600 mL/day for 5 days prior to surgery
Anesthesia:	Spinal blockage	Spinal blockage
Antibiotic prophylaxis:	Kefazolin: 2 g during anesthesia induction followed by 1 g every 8 h for 48 h.	Kefazolin: 2 g during anesthesia induction followed by 1 g every 8 h for 48 h.
Drains and catheters:	Not used	Not used.
Intravenous fluids:	Intra-operative: 5 to 10 mL of crystalloids/kg/h. Postoperative course: 0.9 % saline solution, 30 to 40 mL/kg/day, until the 2^nd^ postoperative day.	Intra-operative: 5 to 10 mL of crystalloids/kg/h. Postoperative course: 1000 mL of Ringer’s solution for 24 h.
Anti thrombotic prophylaxis:	20 mg of enoxaparin immediately post-operative (6 h after anesthetic block) and 40 mg/day from the 1^st^ until the 35^th^ postoperative day. Use of medium leg compression stockings	20 mg of enoxaparin immediately post-operative (6 h after anesthetic block) and 40 mg/day from the 1^st^ until the 35^th^ postoperative day. Use of medium leg compression stockings
Early feeding:	Diet at will starting 6 h after surgery	Diet at will starting 2–4 h after surgery
Mobilization:	Sit up and walk on the 1^st^ postoperative day.	Sit up and walk the same day as surgery.

### Anesthesia and operative techniques

For antibiotic prophylaxis, 2 g of intravenous kefazolin was administered at the time of anesthesia induction. For preoperative, sedation intravenous midazolam (0.03 to 0.1 mg/kg) and propofol (30 to 60 mcg/kg/min) were given. Neither group received general anesthesia. All subjects received epidural neuro-axis blockage at the L3-L4 space with ropivacain 7.5 to 10 % (150–200 mg) and morphine chlorate 2 mg (30 to 50 micrograms/kg). The hip was accessed surgically using an anterior-lateral approach. For all patients, we used a hybrid type of prosthesis for total arthroplasty (cementation of the femoral component and fixation of the acetabular component by means of pressure fitting of the implant to the bone and the addition of screws to the acetabular cup when necessary).

### Carbohydrate drink

Patients belonging to the ACERTO group received a drink (200 mL) containing water plus 12 % maltodextrin 2 h before the induction of anesthesia.

### Immune supplementation therapy

The patients in the ACERTO group received an immune supplement containing arginine, ω-3 fatty acids, nucleotides and vitamins (Impact; Nestlé; Sao Paulo, Brazil). Formula: proteins: 23 % (77 % calcium caseinate, 23 % arginine); carbohydrates: 52 % (100 % maltodextrin); lipids: 25 % (68 % fish oil; 20 % medium-chain triglycerides and 12 % corn oil), vitamins and electrolytes for five days prior to surgery, with a daily dosage of three 200 mL drinks (600 mL per day in total).

### Blood samples

Blood samples were collected preoperatively at anesthesia induction and on the 2^nd^ postoperative day and assayed for CRP.

### Criteria for hospital discharge

Discharge was ordered by an orthopedic surgeon if all five of the following conditions were met: 1) patient is confident and willing to go home, 2) no pain or pain controlled by oral analgesics, 3) walking alone or with minimal help, 4) no fever or signs of postoperative infection and 5) accepting normal oral diet.

### Outcome variables

The main outcome variable was the postoperative LOS. As a secondary outcome variable we compared the evolution of serum C-reactive protein over time.

### Statistical analysis

The calculation of the sample size was based on the premise that the intervention with the ACERTO Project protocol would reduce the hospital LOS by 50 %. Assuming a β error (type II) of 20 % and a α error (type I) of 5 %, sample size calculations indicated 15 patients per group would be sufficient for this study. We used paired and unpaired Student’s t-test when samples had homogeneous variances or the Mann-Whitney test or the Wilcoxon test if samples had heterogeneous variances. CRP levels obtained at two different times were compared by repeated measures ANOVA. Categorical variables were compared by the chi square test or Fisher exact test. Statistical analyses were performed using SPSS 17.0 software and the null hypothesis rejection was fixed at *P* = 0.05 (α = 5 %).

## Results

**Fig. 1:**
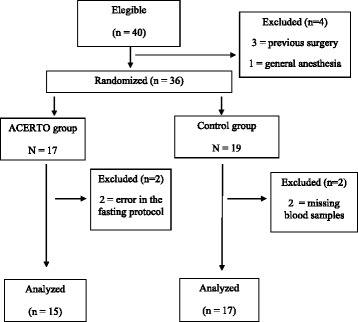
Flowchart of the study

The demographic and clinical characteristics of the two groups are displayed in Table 2 and indicated homogeneity between the two groups. Mean body mass index was 27 (20–31 kg/m^2^) without difference between groups. The flowchart of the study can be seen in Fig. 1. Forty patients were eligible, 36 were randomized and 32 were analyzed (15 in ACERTO group and 17 in control group). There were no deaths. No patients experienced bronchial-aspiration during the anesthesia induction. The duration of the operations ranged from 80 to 125 min (mean = 107 min) for the control group and 85 to 128 min (mean = 103 min) for the ACERTO group (*P* = 0.46). No patients in either group developed a postoperative infection during 60 days of follow-up, required another surgical intervention, were re-admitted in the hospital or require a blood transfusion in the two groups. The ACERTO group received significantly less intravenous crystalloid fluids than controls.

**Table 2:** Preoperative demographic and clinical data of the two study groups of patients who underwent total hip arthroplasty

Variables	Control Group		ACERTO Group		p^a^
	(*n* = 17):		(*n* = 15)		
	n(%) or Mean ± DP		n(%) or Mean ± DP		
Age (years)	58	±17	57	±12	0.92
Sex					
Male	8	47 %	8	53 %	0.72
Female	9	53 %	7	47 %	
Comorbidities^b^					
Yes	10	59 %	12	80 %	0.20
No	7	41 %	3	20 %	
Nutritional					
Assessment					
Malnourished	5	29 %	2	13 %	0.40
Eutrophic	12	71 %	13	87 %	
Immune supplement					
Yes	0	0 %	15	87 %	<0.01
No	17	100 %	0	13 %	
Fasting time (h)	9.52	±1.54	2	±0	<0.001
Hb (g/dL)	13.35	±1.10	12.95	±1.39	0.38
CRP (g/L)	10.24	±5.09	10.13	±6.38	0.96

*SD* standard deviation; *n* number of patients; *VHS* velocity of hemo- sedimentation; *CRP* C-reactive protein; *Hb* hemoglobin

^a^Chi square, Fisher Exact Test and Wilcoxon t test

^b^Diabetes mellitus, arterial hypertension, or chronic obstructive pulmonary disease

### CRP assay

**Fig. 2:**
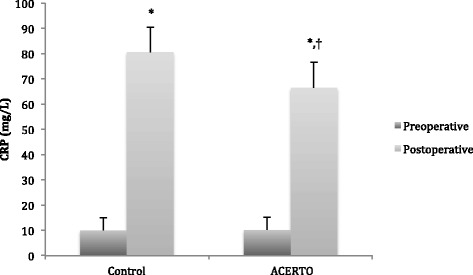
Preoperative and postoperative C-reactive protein (CRP) values in the 2 study groups. Each bars depicts the mean and SD of the CRP values. *, *p* < 0.05 postoperative versus preoperative CRP values (repeated measures ANOVA). †, *P* < 0.01 control versus ACERTO group, Student’s T Test

The findings of the CRP assays are displayed in in Fig. 2 and Table 3. Preoperative CRP values were similar between groups (ACERTO group = 10.2 [6.3] mg/L vs Control group = 10.2 [5.1] mg/L; *p* > 0.05). Although CRP values increased over time in both groups, on the 2^nd^ postoperative day, they were greater in the controls than in the ACERTO group (80.6 [10.9] vs 66.5 [16.4] mg/L, *p* < 0.01).

**Table 3:** Comparison of laboratory results in the two study groups by time period

Variable	Control group		ACERTO group		P*
	Mean ± SD	Median	Mean ± SD	Median	
Hemoglogin (9/dL)					
Preoperative	13.3 ± 1.1	13.5	12.9 ± 1.4	13.0	0.38
2^nd^ PO day	10.3 ± 1.1	10.2	10.2 ± 1.2	10.2	0.78
CRP (mg/L)					
Preoperative	10.2 ± 5.1	8	10.1 ± 6.4	8	0.96
2^nd^ PO day	80.6 ± 10.9	79	66.5 ± 16.5	66	<0.01

Student’s T test

*CRP* C-reactive protein; *SD* standard deviation

* *p* < 0.05 postoperative versus preoperative CRP values (repeated measures ANOVA)

### Length of stay

**Fig. 3:**
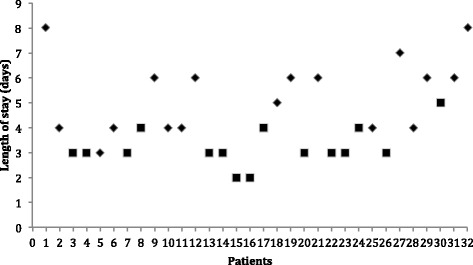
Length of postoperative stay of each patient belonging to the ACERTO group (square) and Control group (diamond). *P* < 0.01

The patients of the ACERTO group (median = 3 days, range 2–5 days) stayed a median of 3 days less (*p* < 0.01) than the controls (median = 6 days, range 3–8 days). Figure 3 shows the LOS of each individual in the two groups.

## Discussion

This study’s findings show that the use of the ACERTO protocol plus preoperative immune nutrition was advantageous for these patients who underwent elective THA. For ACERTO group, the increase of postoperative CRP levels was mild and the patients in this group were discharged earlier. Although this is a pilot study with a small number of patients, the two groups were similar, implying that these findings are reliable. Moreover, the surgical team, the type of operation, the prosthetic device, and the hospital were always the same, which implies internal validity of this comparison.

No other study in Brazil has investigated the modern precepts of the ACERTO project (shortened fasting, restriction of perioperative hydration, early mobilization and preoperative immune nutrition) for elective THA. Our findings suggest that the ACERTO protocol is safe for THA patients and does not increase the risk of bronchial aspiration during anesthesia induction, which is relevant for other types of surgeries [4, 12]. Therefore, the main implication of the findings of this pilot study is the urgent need for more study of the ACERTO protocol in orthopedic surgery because the protocol seems safe, reproducible and beneficial to patients undergoing elective THA.

The prescription of an overnight preoperative fasting is very common. However, the evidence behind this traditional protocol is weak [13]. The actual length of postoperative fasting may be greater than prescribed, which results in fasting times greater than 12 h [14]. Substantial evidence has shown that a 6–8 h fast for solids and 2–3 h for carbohydrate drink is safe and recommended by several societies of anesthesiologists [15, 16]. Prolonged preoperative fasting initiates a metabolic response that may increase the organic response to trauma resulting in increased insulin resistance [17]. The metabolic effect of prolonged fasting and trauma may impair the recovery of surgical patients [18]. Some studies of abdominal surgery have shown that the postoperative LOS may be reduced by shortening preoperative fasting [19]. Our findings agree with this recent literature; we did not observe anesthetic complications, and the ACERTO group recovered faster.

THA patients frequently have minor to mild preoperative inflammation depending on the type of joint disease. After surgery, inflammatory markers such as CRP and procalcitonin increase due to the acute-phase response to trauma. CRP is one of the best biochemical markers for detecting the postoperative inflammatory systemic syndrome after abdominal surgery [20]. CRP has an advantage over procalcitonin; it is less expensive. CRP is also used to monitor the postoperative course of surgical trauma following orthopedic implants and to detect prosthetic infection [21]. Postoperative recovery in orthopedic surgery is directly associated with the magnitude of CRP or other acute-phase mediators; the lower the CRP levels the faster postoperative recovery [22]. The lower CRP postoperative values of the ACERTO group were probably due to several components of the multimodal protocol, such as the shorter period of preoperative fasting [23, 24] and the use of a preoperative immune enhancing supplement [25, 26]. Immune supplements containing arginine, omega-3 fatty acids and nucleotides may improve wound healing and decrease inflammation postoperatively and are currently recommended for major abdominal operations [27]. Unfortunately, we have not found reports of randomized trials of the use of preoperative immune nutrition in THA surgery. Thus, these new data provide a relevant contribution to the orthopedic surgery literature; consequently, further studies are warranted.

Early postoperative feeding after surgery is safe and associated with rapid recovery. However, most of the investigations on early postoperative feeding were conducted after abdominal surgery [28]. Although lack of similar randomized trials in orthopedic surgery hinders comparisons, based on the available reports, resuming feeding soon after surgery is safe and may decrease the volume and duration of intravenous fluids required. On the other hand, the maintenance of *nil per os* during one or two days postoperatively seems currently unacceptable. The longer the postoperative fasting time the greater the need of intravenous crystalloid therapy. Administration of traditional volumes of intravenous crystalloid fluids may impair recovery and may lead to more frequent postoperative infections [29]. Therefore, as soon as possible, the oral route should be initiated and intravenous therapy discontinued. The patients submitted to the ACERTO protocol received less volume of intravenous fluids, and this measure, in addition to others, probably enhanced recovery in these patients.

The results of this study showed that patients submitted to the ACERTO multimodal protocol had shorter LOS and a smaller postoperative rise in their CRP levels. However, because this is a pilot study with few patients, these findings should be considered with caution. Further randomized trials to investigate the benefits of this protocol or other enhancing recovery protocols in either THA or other elective orthopedic surgery are warranted.

## Conclusion

The present findings allow us to conclude that the use of the ACERTO protocol plus preoperative immune nutrition in elective THA may shorten the postoperative length of hospital stay and reduce the acute-phase inflammatory response.

### Availability of supporting data

The data set supporting the results of this article is included in an additional file.

## Abbreviations

ACERTO: acceleration of postoperative recovery
ASA: American Society of Anesthesiologists
CHO: carbohydrates
CRP: C-reactive protein
Hb: hemoglobin
LOS: length of stay
THA: total hip arthroplasty
