# Erratum to: ‘Multimodal perioperative care plus immunonutrition versus traditional care in total hip arthroplasty: a randomized pilot study’

**DOI:** 10.1186/s12937-016-0176-7

**Published:** 2016-05-18

**Authors:** Miguel Aprelino Alito, José Eduardo de Aguilar-Nascimento

**Affiliations:** 1 Brazilian Society of Orthopedics and Traumatology, Cuiabá, MT Brazil; 2 Federal University of Mato Grosso, Cuiabá, Brazil; 3 UNIVAG, Varzea Geande, Brazil; 4 Rodovia Helder Candia, Cond. Country casa 15, Cuiabá, 78048-150 Brazil

Unfortunately, the original version of this article [1] contained an error. There is an error in Fig. 2 of this article. It has been labelled incorrectly and the groups were inverted. The correct figure with correct labels has been included here.

**Fig. 2:**
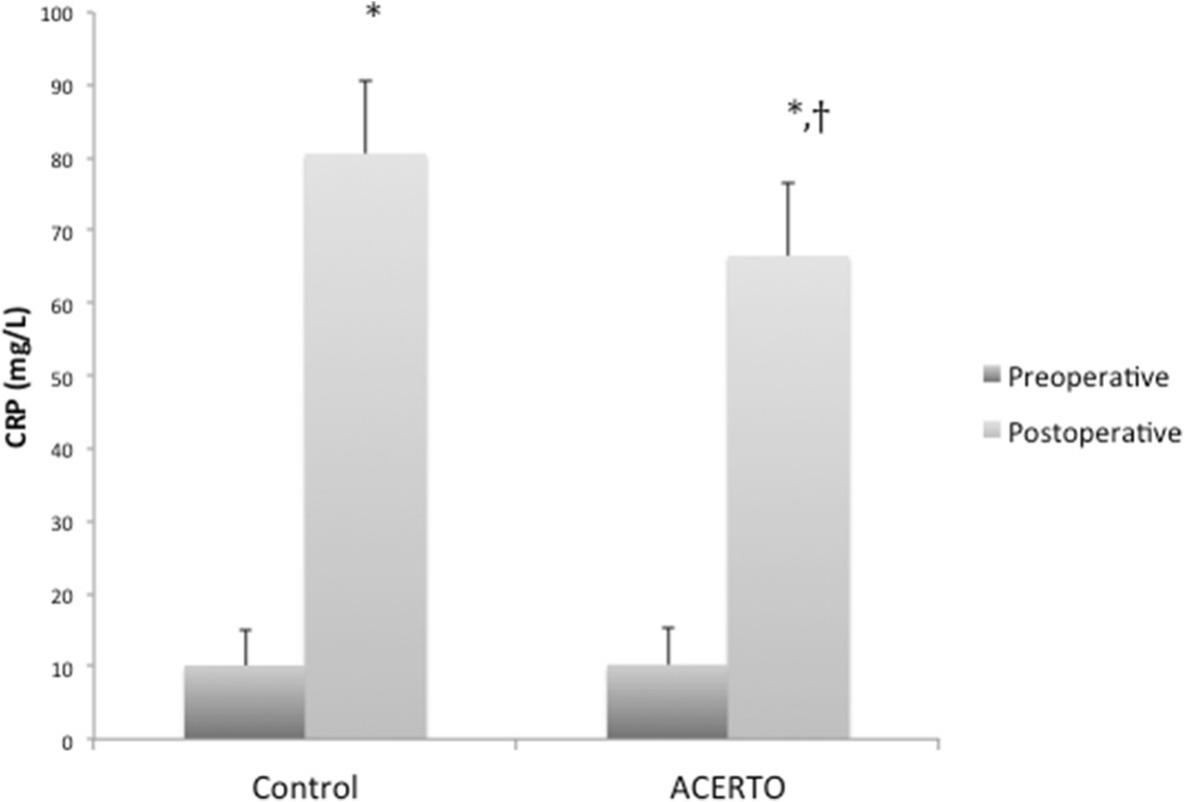
Pre- and postoperative values of C-reactive protein (CRP) in the two groups. *P < 0.05 versus preoperative. ^†^ *P* < 0.05 versus Control group
